# Association between use of systematic reviews and national policy recommendations on screening newborn babies for rare diseases: systematic review and meta-analysis

**DOI:** 10.1136/bmj.k1612

**Published:** 2018-05-09

**Authors:** Sian Taylor-Phillips, Chris Stinton, Lavinia Ferrante di Ruffano, Farah Seedat, Aileen Clarke, Jonathan J Deeks

**Affiliations:** 1 Warwick Medical School, The University of Warwick, Coventry CV4 7AL, UK; 2 Institute of Applied Health Research, College of Medical and Dental Sciences, University of Birmingham, Edgbaston, Birmingham, UK; 3 National Institute for Health Research (NIHR) Birmingham Biomedical Research Centre, College of Medical and Dental Sciences, University of Birmingham, Edgbaston, Birmingham, UK

## Abstract

**Objective:**

To understand whether international differences in recommendations of whether to screen for rare diseases using the newborn blood spot test might in part be explained by use of systematic review methods.

**Design:**

Systematic review and meta-analysis.

**Data sources:**

Website searches of 26 national screening organisations.

**Eligibility criteria for study selection:**

Journal articles, papers, legal documents, presentations, conference abstracts, or reports relating to a national recommendation on whether to screen for any condition using the newborn blood spot test, with no restrictions on date or language.

**Data extraction:**

Two reviewers independently assessed whether the recommendation for or against screening included systematic reviews, and data on test accuracy, benefits of early detection, and potential harms of overdiagnosis.

**Analysis:**

The odds of recommending screening according to the use of systematic review methods was estimated across conditions using meta-analysis.

**Results:**

93 reports were included that assessed 104 conditions across 14 countries, totalling 276 recommendations (units of analysis). Screening was favoured in 159 (58%) recommendations, not favoured in 98 (36%), and not recommended either way in 19 (7%). Only 60 (22%) of the recommendations included a systematic review. Use of a systematic review was associated with a reduced probability of screening being recommended (23/60 (38%) *v* 136/216 (63%), odds ratio 0.17, 95% confidence interval 0.07 to 0.43). Of the recommendations, evidence for test accuracy, benefits of early detection, and overdiagnosis was not considered in 115 (42%), 83 (30%), and 211 (76%), respectively.

**Conclusions:**

Using systematic review methods is associated with a reduced probability of screening being recommended. Many national policy reviews of screening for rare conditions using the newborn blood spot test do not assess the evidence on the key benefits and harms of screening.

## Introduction

Worldwide, the conditions screened for by the newborn blood spot test vary widely,^1^ ^2^ with the number ranging from five to 60 on screening panels.^3^ ^4^ Effective screening programmes can save lives, whereas ineffective programmes can do more harm than good—for example, through overdiagnosis, the physical and psychological consequences of false positive test results, and opportunity costs for the healthcare system. It is not known whether the differences between countries result from genuine differences in disease prevalence or healthcare systems and priorities, or from differences in the evidence review process used to generate policy,^5^ in particular the use of systematic reviews.

Since Wilson and Jungner produced their World Health Organization report on screening in 1968, there has been a divergence in the methods used internationally for policy making about screening.^6^ In Denmark, Finland, France, Germany, Italy, the Netherlands, Sweden, the UK, Australia, and New Zealand, national and regional organisations have updated and amended the Wilson and Jungner principles to fit their local context and to use their own versions to make policy recommendations and decisions about screening.^7^ In the United States, the US Preventative Services Task Force has developed an analytical framework that is adapted to the particular circumstances of each review.^8^ This includes three key elements that might determine the balance of benefits and harms from implementing screening for a condition: test accuracy for detecting the condition of interest; the benefit of early detection, and therefore treatment after screening compared with later detection following symptoms; and the extent of overdiagnosis, one of the main harms of screening owing to the detection of disease that would never have caused symptoms within someone’s lifetime.

We analysed national policy making decisions about which conditions to screen for using the newborn blood spot test to determine whether systematic reviews were undertaken and if this was associated with the final recommendation of whether to implement screening. We also scored the extent to which each decision making process considered test accuracy, the benefit of early detection, and overdiagnosis, and investigated associations with the final decision.

## Methods

### Search

We searched the websites of national policy making organisations for all documentation related to the newborn blood spot test (see appendix 1 for organisations). A previous systematic review was used to identify these organisations.^7^ We asked a panel of international screening experts to identify any further documentation, and we searched website databases of WHO, the European Council, the European Commission, and the European Observer. From the included documentation, we extracted and synthesised data describing the process of reaching decisions for every condition considered for inclusion on the newborn blood spot screening panel, with no restrictions on date or language.

The initial search for this review was conducted on the websites of these national organisations on 18 September 2015 using search terms for newborn blood spot screening and the conditions included by the American College of Medical Genetics (see appendix 2 for full search terms). We emailed each organisation and country experts requesting any further documentation on newborn blood spot screening. If either referred us to associated but different organisations, we searched those websites using the same search terms between 18 September 2015 and April 2016 (for example, in the US we searched the Preventative Task Force website and found that recommendations for the blood spot test are made by the Advisory Committee on Heritable Disorders in Newborns and Children. Similarly, after contacting the Ministry of Social Affairs and Health in Finland, we found that relevant reviews are on the Finnish HTA website). Overall, we searched the websites of 26 organisations.

### Inclusion criteria

Two reviewers independently assessed each item against the inclusion criteria, with disagreements resolved by consensus. The inclusion criteria were:

*Source of documents*—only information from national policy making organisations was included. We excluded recommendations by state or regional organisations unless endorsed by a national policy making organisation, and recommendations by clinical societies or other groups unless they were explicitly used to underpin national policy decisions.

*Type of document*—we included all journal articles, papers, legal documents, presentations, conference abstracts, or reports from the website of the organisation and all those obtained through personal communication with policy makers, officials, and researchers in all included countries. We did not include patient information.

*Language*—there were no restrictions on language. For documents not in English we used automated translation software, with formal translation by native speakers if further clarity was needed.

*Subject of documents*—we included material on whether to start or stop screening or material that evaluated the effectiveness of current or proposed screening programmes for any condition using the newborn blood spot test. If we also found reviews of conditions for that country, we included documents describing standards for national evidence review processes for screening.

*Method of reaching recommendation*—we included recommendations produced using all methods, including evidence from systematic reviews, expert panels, or any approach that resulted in a recommendation or decision or described why or how a decision was made.

### Data extraction

Two reviewers independently extracted data, with disagreements resolved by consensus and involvement of a third reviewer if necessary (see appendix 3 for data extraction sheet). Data extraction was carried out in two steps. Firstly, we recorded whether any of the review documentation included a systematic review. The criteria for defining a systematic review were inclusive; we required either two parts of the search strategy (for example, search terms, databases, dates) to be described or any details of systematic evidence selection after a search (for example, inclusion criteria, PRISMA flow chart) to be described (table 1). We were also inclusive about the question posed by the systematic review, which could address any aspect of the evidence relating to whether or not to screen for a condition, including benefits of early detection through screening, disease prevalence, test accuracy, effects of false positive test results, overdiagnosis or any other harm, and clinical course of the condition.

**Table 1:** Criteria for defining whether each country undertook a systematic review for each condition, with examples

Country	Condition	Systematic review used	Rationale for classification
Netherlands	Carnitine acylcarnitine translocase deficiency	No	No methods given, but likely expert consensus. Section 1.3.4 states “the committee believes that this disease should be classified in Category 1^79^” (category 1 refers to conditions that the committee considered as qualifying for inclusion in the newborn screening programme)
Denmark	Multiple carboxylase deficiency	No	Section 4 states: “[we] assessed the conditions selected for additional analysis, which was based on a review of original literature including treatment options, screening potential and experience.”^82^ No further details of the review process were provided
Canada	Phenylketonuria	Yes	Section 17 outlined the review methods, and included: source searched (Medlline only), search term (phenylketonuria), and date limit.^95^ Meets criterion for describing two parts of the search strategy
UK	Long chain 3-hydroxyacyl-CoA dehydrogenase deficiency	Yes	“Chapter 5 provides a methodology for the systematic review.” This included the search strategy, resources searched (electronic databases and reference lists of identified articles), search terms, date limit, language restrictions, and number of reviewers; and the inclusion and exclusion criteria.^22^ Meets both criteria for defining a systematic review because at least two parts of the search strategy and inclusion criteria were described

Criteria for defining a systematic review: A: describes two parts of the search strategy (eg, search terms, databases, dates), or B: describes any details of systematic evidence selection after a search (eg, inclusion or exclusion criteria, numbers at abstract and full text sift, PRISMA flow diagram). Each country was defined as having undertaken a systematic review for each condition if either criterion A or B, or both, were met.

The review topic could be about any aspect of screening for the disease under consideration (eg, benefits of early detection through screening, disease prevalence, test accuracy, effects of false positive test results, overdiagnosis or any other harm, clinical course).

Secondly, we assessed three key elements characterising the main benefits and harms of screening: test accuracy, benefits of early detection through screening, and overdiagnosis. These characteristics were selected on the basis of our review of published frameworks for test evaluation^9^ ^10^ ^11^ ^12^ to identify all mechanisms recognised to affect patient health as a result of undergoing testing or taking part in a screening programme.

Table 2 details the scoring system for the assessment of evidence related to the three key elements. We measured whether and how the evidence was assessed; not what the evidence showed about that particular condition. A score of zero means that the element was not mentioned in the documentation, with increasing scores up to a score of 5 indicating greater and more systematic use of evidence and increasing assessment of internal and external validity. A score of ≥3 for any of the three key elements indicates that a systematic review was used for that recommendation. In some cases a systematic review was used and recorded as such but the review did not cover test accuracy, benefit of early detection, or overdiagnosis. In such cases, the evidence would score <3 for these three key elements in the secondary analyses but was still coded as a systematic review in the primary analysis (meta-analysis).

**Table 2:** Scoring system for assessment of evidence for test accuracy, benefit of early treatment, and overdiagnosis

Score*	Definition	Examples		
		Test accuracy	Benefit of earlier treatment	Overdiagnosis
0	Not considered at all	USA, American College of Medical Genetics recommendation to screen for 3-hydroxy-3-methyglutaric aciduria “Screening test: MSMS [tandem mass spectrometry]. Reported in 1990 [references given]” (the references provided refer to how to undertake testing using MS/MS, but provide no details on test accuracy^13^)	USA, argininemia: “Treatment is expected to reduce neurological dysfunction [references given].” References refer to treatment effectiveness not benefit of earlier treatment (after screen detection) over later treatment (after symptomatic detection)^13^	Overdiagnosis not mentioned using any form of wording, including asymptomatic phenotypes, penetrance, and any description of people remaining symptom-free to adulthood
1	Considered in some way (mentioned in at least one document once)	Netherlands, tyrosinemia type I: “It is possible to make the test specific for tyrosinemia type I and greatly reduce the number of false-positives by also measuring the amount of succinyl acetone in the blood specimen [no reference given]”^14^	USA, congenital hypothyroidism: “Some evidence that early intervention optimizes individual outcomes [no reference given]”^13^	USA, 3-methylcrotonyl-CoA carboxylase deficiency: “since newborn screening with MS/MS [tandem mass spectrometry] began, many individuals have been identified with the analytes associated with the condition but without apparent clinical manifestations [no reference given]”^13^
2	Measured in some way (at least one study or source cited, and for test accuracy at least some numerical estimate given) or acknowledged that data do not exist yet	New Zealand, economic model of screening for severe combined immunodeficiency: table 4 model assumptions “test sensitivity 0.999, test specificity 0.996 [reference given]”^15^	New Zealand, nomination form for removal of 3-methylcrotonyl-CoA carboxylase deficiency 3MCC from the screening panel: “RCTs [randomised controlled trials] are not possible in newborn metabolic screening due to the low incidence of the disorders, and the time period required to generate a statistically significant number of cases in the screening arm versus the control arm. Case studies suggest screening is not effective in reducing mortality or morbidity [reference given]”^16^	Denmark, biotidinase deficiency: “It is unclear whether asymptomatic children with partial biotinidase deficiency need treatment [references given]”^17^
3	Investigated using systematic methods of collecting evidence (score if detail two parts of search strategy or any details of evidence selection methods)	Spain, findings of a systematic review of biotidinase: “Therefore sensitivity and specificity of the test is estimated at 100% and 99.994%, respectively. These results are very similar to those presented in Kwon & Farrel, 2000 [reference given]”^18^	Canada, systematic review of cystic fibrosis (CF): “Before any screening program is implemented there should be good evidence that people identified in the presymptomatic phase do better than those in whom a diagnosis is made because of symptoms . . . Several cohort studies of screened and unscreened subjects have suggested that early diagnosis does make a difference. In one study [26] in the Netherlands, 88% of screened children but only 60% of unscreened children were still alive at age 11 years. In an earlier study by the same group, screened children were found to have better clinical scores at age 8 years than did unscreened children with CF, but the differences in chest x-ray films, heights and weights were not statistically significant [reference given]”^19^	France, systematic review of medium-chain acyl-CoA dehydrogenase deficiency: “Screening results in the United States, Germany and Australia have revealed the presence of a relatively frequent mutation which was not found in patients exhibiting clinical symptoms [references given]. Studies in vitro have demonstrated that this mutation is associated with a reduction in the enzymatic activity which may not necessarily have any clinical significance [reference given]”^20^
4	Systematic review and mention external validity (generalisability to local context) or internal validity (bias or confounding) of evidence or hierarchy of evidence	Spain, systematic review of classic galactosemia: “sensitivity of 100% and a specificity of 99.9% in all programs, although these data should be interpreted with caution in the absence of studies to conduct a verification of negative cases [reference given]”^21^	UK, systematic review of maple syrup urine disease: “Other authors provide shorter case-history approaches to identification of improved clinical outcomes from screen detected patients. These include [references given] all of whom compare small numbers of pre-symptomatically detected versus clinically detected cases but without construction of comparative cohorts”^22^	USA, systematic review of Krabbe disease: “Of the seven high-risk cases detected in New York (Table 6), two were considered EIKD [early infantile Krabbe disease] and referred for HSCT [hematopoietic stem cell transplant] because of their GALC [galactosylceramidase] genotypes and the early signs of neurologic disease. One of these patients was homozygous for the 30-kb deletion mutation, while the other patient was heterozygous for the 30-kb deletion and a novel mutation. Dr. Wenger reports that the five remaining children who screened high risk had genotypes considered to put them at a low risk for early onset of disease. Dr. Caggana and Dr. Orsini state that two of these children were lost to follow-up and three are being followed on a quarterly basis by a neurologist. One of these children is known to be asymptomatic and the other two are assumed to be asymptomatic as Dr. Caggana and Dr. Orsini have not heard otherwise^23^
5	Systematic review and assessed using formal quality assessment	No examples found	Belgium, systematic review of CF provides full quality assessment of the studies in an appendix, with summary: “The studies performed to support CF NBS [newborn screening] is not as strong as one might expect, knowing that there are still two large randomized trials (RCTs) [that] were designed to evaluate CF NBS. The design of the UK RCT (1985-1989) was substandard and this study was therefore not retained in a recent Cochrane review. The Wisconsin RCT (1985-1995) did have a proper design and demonstrated a significant advantage of CF NBS in the field of nutrition and growth (weight and length). However, in [relation to] lung function, no benefit from CF NBS could be demonstrated”^24^	No examples found

* Scores are cumulative—for example, a score of 3 can only be achieved if meeting all criteria to score 1, 2, and 3.

Test accuracy determines how many people are detected early with true positive test results and how many are potentially harmed by false positive results. The scoring system refers to whether there is an accurate test, which can include any test accuracy metrics such as sensitivity, specificity, and positive or negative predictive value. Consideration of the existence of a test is a necessary prerequisite but does not form part of the scoring system. The benefit of early detection leading to early treatment is the primary mechanism through which screening provides benefit. The scoring system refers specifically to the benefit of early treatment, not whether there is an effective treatment, which is also a prerequisite. Overdiagnosis in this context is defined as detection of disease at screening that would never have produced symptoms within someone’s lifetime. We were inclusive in the language used to describe overdiagnosis, including asymptomatic phenotypes, penetrance, and any description of people remaining symptom-free to adulthood.

### Statistical analysis

Cohen’s κ was used to calculate inter-reviewer reliability for judgments of whether a systematic review was used, scores for the test accuracy, benefits of early detection, and overdiagnosis, and whether screening was recommended, with linear weighting when more than two categories existed, and interpretation according to Landis and Koch.^25^ We report proportions of included decisions that used systematic review methods; the methods used to assess test accuracy, benefit of early detection, and overdiagnosis (graphs show distribution of scores); and the final recommendation tabulated by country. To determine whether the patterns observed were purely historical we repeated the analysis including only policies since 2012.

We computed the odds ratio for recommending screening for each condition if a systematic review was used compared with recommending screening if a systematic review was not used. To get an overall estimate of the impact of using systematic reviews on policy formation of recommendations, we meta-analysed odds ratios across conditions. This stratified approach removes the confounding effect of clinical condition. Only conditions where there were discrepancies in recommendations (ie, at least one recommendation for and one recommendation against screening) and in methods (ie, at least one recommendation with systematic review evidence and one without) could contribute to this comparison and were included in the meta-analysis. We calculated an overall effect estimate using Mantel-Haenszel fixed effects meta-analysis with a 0.1 zero cell correction.^26^ ^27^ The analyses were repeated with no and other values of zero cell correction (0.5, 0.01, 0.001), using the DerSimonian and Laird random effects method with zero cell correction 0.5, and the Peto method.^27^ We tested for heterogeneity using Cochran’s Q and described its magnitude using the I^2^ statistic. All analyses used Stata version 13.

Spearman correlation was used to univariately assess the relation between policy recommendations and the rigor of methods used to assess test accuracy, the benefits of early detection and treatment, and the risks of overdiagnosis (only systematic reviews of conditions for which there were recommendations both for and against screening were included in this analysis).

### Patient involvement

No patients were involved in setting the research question or the outcome measures, nor were they involved in developing plans for design or implementation of the study. No patients were asked to advise on interpretation or writing up of results. We will work with patients and members of the public to help disseminate findings to appropriate audiences.

## Results

### Description of evidence

**Fig 1:**
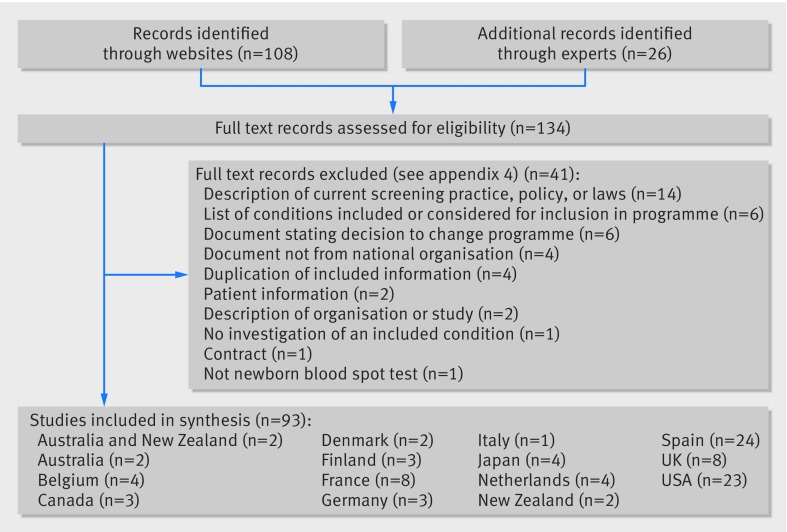
Flow of documents through study. One paper was included from Italy, but no national decisions in the analysis, because one paper that will be used in part to underpin the national decisions has been published, but the national review process is incomplete and recommendations are yet to be made

We identified 134 policy documents (fig 1), 108 of which were from screening organisation websites and 26 referred from experts. Overall, 41 documents were excluded. Reasons for exclusion were: description of current screening practice, policy, or laws; list of conditions included or considered for inclusion in programme; document stating decision to change programme; document not from national organisation; duplication of included information; patient information; description of organisation or study; no investigation of an included condition; contracts; and not newborn blood spot test (see appendix 4 for references of exclusions with reasons). After exclusions, 93 reports remained.^13^ ^14^ ^15^ ^16^ ^17^ ^18^ ^19^ ^20^ ^21^ ^22^ ^23^ ^24^ ^28^ ^29^ ^30^ ^31^ ^32^ ^33^ ^34^ ^35^ ^36^ ^37^ ^38^ ^39^ ^40^ ^41^ ^42^ ^43^ ^44^ ^45^ ^46^ ^47^ ^48^ ^49^ ^50^ ^51^ ^52^ ^53^ ^54^ ^55^ ^56^ ^57^ ^58^ ^59^ ^60^ ^61^ ^62^ ^63^ ^64^ ^65^ ^66^ ^67^ ^68^ ^69^ ^70^ ^71^ ^72^ ^73^ ^74^ ^75^ ^76^ ^77^ ^78^ ^79^ ^80^ ^81^ ^82^ ^83^ ^84^ ^85^ ^86^ ^87^ ^88^ ^89^ ^90^ ^91^ ^92^ ^93^ ^94^ ^95^ ^96^ ^97^ ^98^ ^99^ ^100^ ^101^ ^102^ ^103^ ^104^ ^105^ ^106^ ^107^ ^108^ Two covered Australia and New Zealand together,^30^ ^33^ two were from Australia,^61^ ^87^ four from Belgium,^24^ ^68^ ^69^ ^105^ three from Canada,^19^ ^37^ ^95^ two from Denmark,^17^ ^82^ three from Finland,^31^ ^59^ ^85^ eight from France,^20^ ^34^ ^35^ ^71^ ^72^ ^99^ ^100^ ^104^ three from Germany,^106^ ^107^ ^108^ one from Italy,^102^ four from Japan,^53^ ^66^ ^67^ ^88^ four from the Netherlands,^14^ ^79^ ^80^ ^81^ two from New Zealand,^15^ ^16^ 24 from Spain,^18^ ^21^ ^32^ ^39^ ^40^ ^41^ ^42^ ^43^ ^44^ ^45^ ^46^ ^47^ ^48^ ^49^ ^50^ ^51^ ^76^ ^77^ ^78^ ^83^ ^86^ ^89^ ^90^ ^91^ eight from the United Kingdom,^22^ ^36^ ^38^ ^52^ ^58^ ^73^ ^84^ ^94^ and 23 from the USA.^13^ ^23^ ^28^ ^29^ ^54^ ^55^ ^56^ ^57^ ^60^ ^62^ ^63^ ^64^ ^65^ ^70^ ^74^ ^75^ ^92^ ^93^ ^96^ ^97^ ^98^ ^101^ ^103^

### Review methods used

Overall, the 93 reports included 104 conditions from 14 countries, giving a total of 276 recommendations (units of analysis). Cohen’s κ for inter-reviewer reliability was 0.91 (near perfect) for whether a systematic review was used, 0.73 (substantial) for test accuracy score, 0.47 (moderate) for benefit of early detection score, 0.62 (substantial) for overdiagnosis score, and 0.97 (near perfect) for the final recommendation of each review.

**Fig 2:**
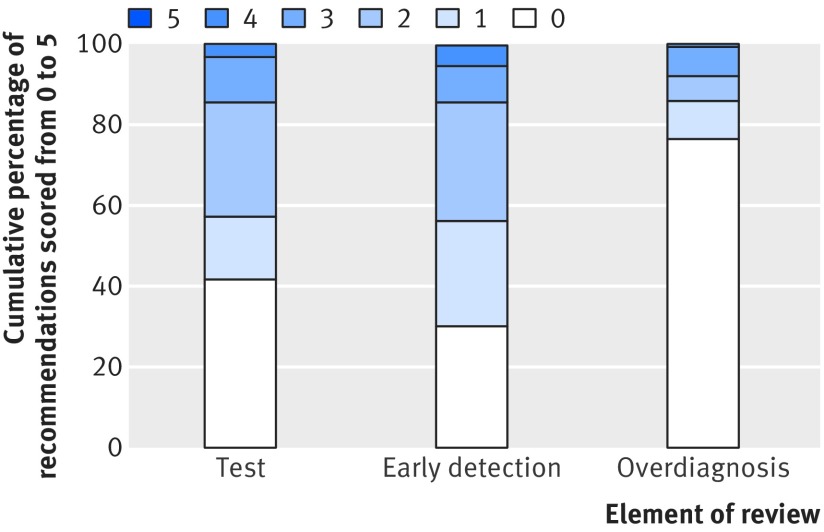
Distribution of scores for evaluating test accuracy, benefits of early versus late detection and treatment, and overdiagnosis. A score of zero indicates that these elements were not considered at all, and 5 indicates that they were assessed using a systematic review with formal quality appraisal

Of the 276 recommendations, 159 (58%) were in favour of screening, 98 (36%) were against screening, and no suggestion was made either way in 19 (7%). Sixty (22%) of the recommendations included evidence from a systematic review. Of the recommendations, evidence for test accuracy, benefits of early detection, and overdiagnosis was not considered in 115 (42%), 83 (30%), and 211 (76%), respectively. Of the 60 recommendations that employed systematic review methods, 21 systematic reviews covered test accuracy, benefits of early detection, and overdiagnosis. Figure 2 shows the full distribution of scores. Similar patterns are observed if only the most recent 154 reviews (from 2012 onwards) are included (see supplemental figure 1). Table 3 shows a full breakdown by country.

**Table 3:** Review methods and decisions for each country

Country	Proportion of decisions, % (No/total No)		Review scores (No*)																			
	Recommended screening	Used systematic review	Test accuracy							Early detection							Overdiagnosis					
			0	1	2	3	4	5		0	1	2	3	4	5		0	1	2	3	4	5
Australia	100 (1/1)	0 (0/1)	0	0	1	0	0	0		0	1	0	0	0	0		0	0	1	0	0	0
Belgium	14 (1/7)	14 (1/7)	0	1	5	1	0	0		0	3	3	0	0	1		4	2	0	1	0	0
Canada	83 (5/6)	67 (4/6)	2	0	0	3	1	0		3	0	1	1	1	0		5	0	1	0	0	0
Denmark	60 (21/35)	0 (0/35)	4	3	28	0	0	0		8	16	11	0	0	0		25	9	1	0	0	0
Finland	0 (0/7)	100 (7/7)	1	4	1	1	0	0		0	7	0	0	0	0		6	1	0	0	0	0
France	33 (1/3)	100 (3/3)	0	0	2	1	0	0		0	0	0	1	2	0		0	2	0	1	0	0
Germany	100 (1/1)	100 (1/1)	0	0	0	1	0	0		0	0	0	0	1	0		0	0	0	1	0	0
Japan	81 (25/31)	0 (0/31)	27	2	2	0	0	0		30	0	1	0	0	0		30	1	0	0	0	0
Netherlands	55 (29/53)	0 (0/53)	21	13	19	0	0	0		18	23	12	0	0	0		48	3	2	0	0	0
New Zealand	13 (1/8)	75 (6/8)	0	5	2	0	0	0		5	1	2	0	0	0		7	0	1	0	0	0
Spain	41 (11/27)	100 (27/27)	6	0	0	20	1	0		3	0	0	22	2	0		10	5	0	12	0	0
UK	75 (6/8)	63 (5/8)	0	0	3	4	1	0		0	2	2	1	3	0		2	0	1	5	0	0
USA	64 (57/89)	7 (6/89)	53	15	15	0	6	0		16	19	49	0	5	0		74	3	10	0	2	0

* Number of included recommendations with each evidence score.

### Association between evidence review methods and recommendations

Of the 60 decisions that included a systematic review, 23 (38%) recommended screening, 29 (48%) recommended not to screen, and eight (13%) made no recommendation either way. The corresponding results for the 216 decisions not based on evidence from a systematic review were 136 (63%), 69 (32%), and 11 (5%).

**Fig 3:**
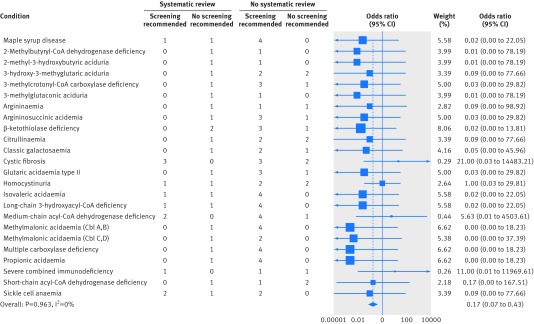
Forest plot of the odds of recommending screening in decisions that included compared with did not include evidence from a systematic review. Overall effect estimate from fixed effects meta-analysis with a 0.1 zero cell correction

The meta-analysis included 24 conditions, each with between two and eight reviews, with 104 reviews in total. The odds of making a decision to recommend screening was lower when a systematic review was used than when no systematic review was used (odds ratio 0.17, 95% confidence interval 0.07 to 0.43, P<0.001; fig 3). Owing to the small sample sizes, little heterogeneity existed between conditions (χ^2^=12.45 (df=23), P=0.96), with none of the total variance due to variability between conditions (I^2^=0%). Sensitivity analyses using different zero cell corrections and meta-analysis methods did not alter the results and were all highly significant (P<0.001), although increasing the zero cell correction did slightly reduce the effect size (see appendix 2).

Review scores for benefits of early detection and overdiagnosis were not statistically significantly correlated with the recommendation of the review, although there was an association between greater consideration of test accuracy in the review and a recommendation against screening (table 4). Confidence intervals were wide, narrowly excluding zero for test accuracy and just overlapping zero for overdiagnosis score.

**Table 4:** Number of reviews recommending screening and no screening by scores for test accuracy, benefit of early detection, and overdiagnosis

Scores	Recommendation		Proportion recommend screening (%)	Spearman correlation coefficient* (95% CI)	P value
	Screening	No screening			
Test accuracy:					
0	41	14	75	−0.17 (−0.33 to −0.01)	0.04
1	10	8	56		
2	27	16	63		
3	10	11	48		
4	2	1	67		
5	0	0			
Benefits of early detection:					
0	27	17	61	−0.06 (−0.22 to 0.11)	0.51
1	23	11	68		
2	30	7	81		
3	5	13	28		
4	4	2	67		
5	1	0	100		
Overdiagnosis:					
0	71	34	68	−0.13 (−0.29 to 0.03)	0.12
1	5	5	50		
2	7	3	70		
3	7	8	47		
4	0	0			
5	0	0			

* Correlation is between scores and whether screening was recommended (only includes reviews of conditions where at least one review recommended screening and one did not).

## Discussion

We assessed whether use of a systematic review affects national decisions on whether to screen for a range of conditions using the newborn blood spot test. After full text review, we included 93 reports assessing a total of 104 conditions across 14 countries, with 276 recommendations. Only 22% of the recommendations were based on evidence from a systematic review. The odds of a decision in favour of screening were lower when a systematic review was used as part of the policy decision (0.17, 95% confidence interval 0.07 to 0.43). The evidence on accuracy of the test was not evaluated in 42% of recommendations. Similarly, the evidence around the benefits of early detection and the potential harm of overdiagnosis were not evaluated in 30% and 76% of reviews, respectively. These elements were actually not mentioned in the review documents, which suggests either lack of evidence review or lack of consideration. For each review, the more thoroughly test accuracy was considered the lower was the probability that screening would be recommended. A weak association was found in the same direction for thoroughness of assessment between both early treatment benefits and overdiagnosis and screening recommendations. However, power was too limited to assess these associations, owing to the low scores creating a floor effect.

### Strengths and limitations of this study

The strengths of this study include the large number of documents extracted using systematic methods, with no restrictions on date, language, or country, and the use of meta-analytical methods to determine whether there was a consistent effect across different conditions thus accounting for confounding by condition. Also we used automated translation software, which enabled broader inclusion criteria, although errors might have occurred in translation. To mitigate this risk, we used formal translation for documents or parts of documents where the automated translation was unclear to reviewers. In addition, the review of grey literature documenting national policy decisions is challenging in itself, particularly on reproducibility since websites change over time. We also contacted every organisation for further documents, but it is possible that more systematic reviews were used than were published or referenced by the national websites of policy makers or identified through personal communication.

Although we found an association between use of systematic reviews and whether or not a screening programme was recommended, the decision on whether to undertake a systematic review might have been driven by country level factors, as four of the 14 included countries always used a systematic review and four never did. Thus it might be possible that use of systematic review methods acted as a proxy for unmeasured country level confounders, so only tentative conclusions can be drawn.

### Comparison with other studies

Previous research has highlighted an underuse of systematic reviews in developing policy guidance for screening programmes. A 2006 study reported that systematic reviews were rarely used in production of WHO guidance, a discovery that initiated a major research effort to incorporate greater use of systematic reviews.^109^ Although the research literature concerning measurement of overdiagnosis is extensive, our study systematically investigated whether consideration of potential overdiagnosis is incorporated into national screening policy decision making. Our main finding, however, was that policy reports that did not utilise systematic review methods were more likely to recommend screening, suggesting that rigorous appraisal exposes the absence or unreliability of available evidence. Indeed, several studies have shown differences between expert opinion and research evidence. One study observed that professional recommendations on treatments for acute myocardial infarction communicated through review articles or textbooks often contradicted the best evidence from meta-analysis of trials available at the time of publication.^110^ An opinion article argued that experts are more likely to overestimate the effectiveness of interventions based on their own clinical experiences.^111^ In fact a systematic review showed that clinicians overestimate the benefits of screening and underestimate the harms.^112^ We consider that quality appraisal in systematic reviewing serves as a mechanism to highlight bias in research studies (often biased away from the null). This might explain why expert policy making groups that use systematic reviews are less likely to recommend screening.

### Policy implications

This study showed that many national policy decisions about whether to screen for conditions using the newborn blood spot test are being made without systematically reviewing the evidence. One reason for this absence is likely to lay in the absence of evidence from randomised controlled trials, which is unavailable for most conditions included in the newborn blood spot owing to their rarity. Indeed, although many countries have developed robust systems for reviewing new screening programmes, we found that they are often not applied when assessing whether to screen for additional rare diseases using the newborn blood spot test. Yet it remains essential to make evidence based policy decisions because once screening programmes are started they are difficult to stop.^12^ When trial evidence is not available, a review of whether to screen for each condition should consider the evidence for each pathway to patient benefit and harm resulting from introducing a screening test, in particular: the test’s ability to discern true disease, any resulting potential for patient harm from overdiagnosis, and the benefits of early detection. Although many reviews considered whether subsequent diagnostic tests and treatments were available to manage screened patients, most did not consider evidence for the screening test’s accuracy, nor whether earlier detection and treatment after screening were beneficial to patients compared with later detection of symptoms and treatment. These three elements are not an exhaustive list of benefits and harms (for example, we did not examine the effect of screening results to other family members); however, there is broad agreement that they are key indicators of effectiveness.^10^ ^11^ We recommend that whenever possible a systematic review of the literature should be undertaken as part of policy decisions on whether to commence screening. Full systematic reviews that assess each key element of a screening programme can be expensive and time consuming—particularly in the absence of trial evidence, and we propose more international collaboration to undertake such reviews. Although the health systems, prevalence, culture, and willingness to pay thresholds might differ by country, the evidence about test accuracy, benefits of early detection, and overdiagnosis are international bodies of evidence, and collating them will be the same regardless of country. Only concerns about applicability will differ.

### Conclusions

Further research is required to understand why policy makers do not employ systematic review methods in their evaluations of evidence. Possible reasons include costs, time, and knowledge and beliefs about systematic reviews.^113^ Undertaking international reviews for conditions across several countries would reduce overall costs. These reviews could be adapted to local populations and prevalence and improve rigour while reducing discrepancies in screening internationally.

**What is already known on this topic**

Decisions about which conditions to screen for using the newborn blood spot test vary widely between countries, despite similar populations and healthcare systems
No systematic assessment has been done of the effect of evidence review methods used by different countries for decision making about screening using the newborn blood spot test

**What this study adds**

Use of a systematic review of the evidence was associated with a reduced probability of screening being recommended
42% of recommendations by national policy making organisations about whether to screen babies for diseases using the newborn blood spot test do not take account of the evidence on test accuracy, 36% do not review evidence about whether early treatment improves health outcomes, and 76% do not consider the evidence around potential harms of overdiagnosis

## Web extra

Extra material supplied by authors

## Notes

Ethical approval: Not required.

Data sharing: The authors are willing to share data by personal communication to ST-P.

Transparency: The lead author (ST-P) affirms that this manuscript is an honest, accurate, and transparent account of the study being reported; that no important aspects of the study have been omitted; and that any discrepancies from the study as planned (and, if relevant) have been explained.
