# Supplementary material for: Association between use of systematic reviews and national policy recommendations on screening newborn babies for rare diseases: systematic review and meta-analysis
Source: BMJ. 2018 May 9;361:k1612. doi: 10.1136/bmj.k1612 (PMC5941220; doi:10.1136/bmj.k1612)
Supplement: Supplementary file 1 — Supplementary information: additional material [file tays040809.ww1.pdf]

Supplemental Figure 1. Distribution of scores for evaluating test accuracy, the benefits of early detection and treatment over late, and overdiagnosis including only recommendations published since 2012. A score of zero indicates that these elements were not considered at all, and 5 indicates that they were assessed using a systematic review was undertaken with formal quality appraisal.

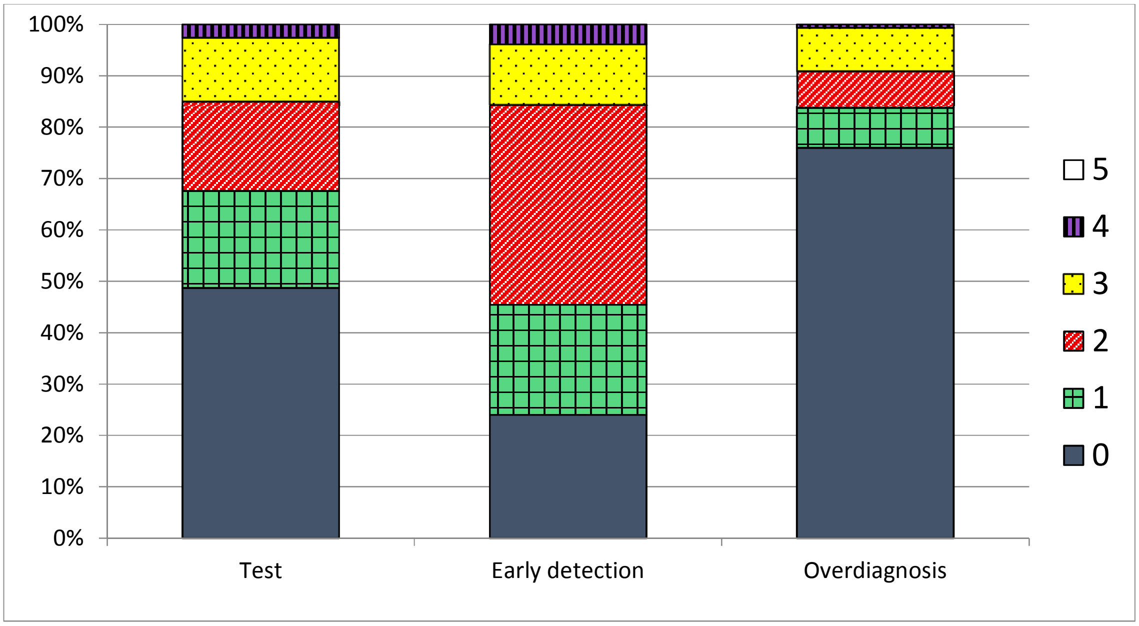

Supplement 2: Meta-analyses using different assumptions

Mantel–Haenszel fixed effects meta-analyses were repeated using different zero cell corrections, and using the Peto method. Results are as follows. The odds of recommending screening were lower when using a systematic review, using a zero cell correction of 0.5 (OR= 0.27, 95%CI 0.14 to 0.55, p<0.0005), of 0.01 (OR= 0.15, 95%CI 0.05 to 0.40, p<0.0005) and of 0.001 (OR= 0.14, 95%CI 0.04 to 0.38, p<0.0005), no zero cell correction (OR= 0.14, 95%CI 0.05 to 0.39, p<0.0005), DerSimonian and Laird random effects meta-analysis with zero cell correction of 0.5 (OR= 0.24, 95%CI 0.11 to 0.53, p<0.0005), or Peto meta-analysis (OR= 0.17, 95%CI 0.08 to 0.39, p<0.0005). Forest Plot for Peto meta-analysis is shown below. Estimate of between-condition variance from the random effects meta-analysis (Tau^2<0.00005).

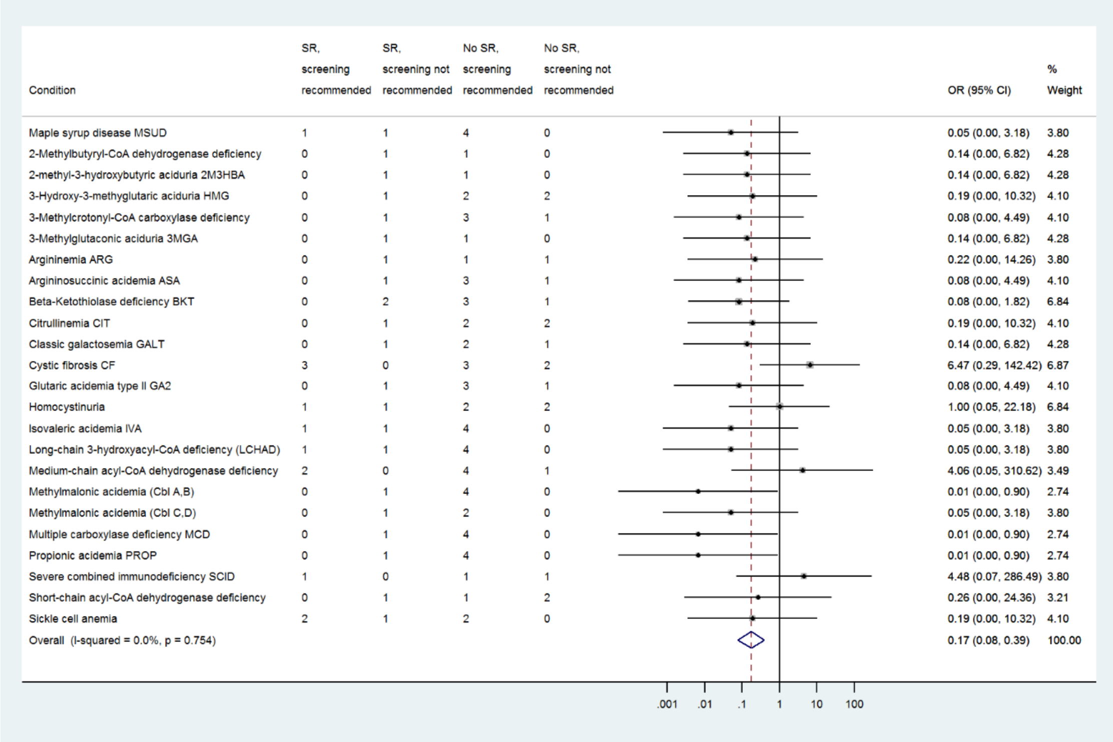

Appendix 1. Websites searched

No	Organisation	Website
	**Initial search**	
1	The Screening Subcommittee, Australian Population Health Development Principal Committee	http://www.cancerscreening.gov.au/
2	Belgium Superior Health Council	https://www.health.belgium.be/en/superior-health-council
3	Canadian Task Force on Preventative Health Care	http://canadiantaskforce.ca/
4	Sundhedsstyrelsen<br>(Danish National Board of Health)	https://www.sst.dk/
5	Social- och hälsovårdsministeriet<br>(National Screening Committee, Ministry of Health and Social Affairs)	http://stm.fi/
6	Haute Autorité de Santé<br>(French High Authority of Health)	https://www.has-sante.fr/
7	Gemeinsamer Bundesausschuss<br>(The Federal Joint Committee)	https://www.g-ba.de/
8	Arbeitsgemeinschaft der Wissenschaftlichen Medizinischen Fachgesellschaften<br>(German Association of the Scientific Medical Societies)	http://www.awmf.org/
9	Osservatorio nazionale screening<br>(National Observatory Screening)	http://www.osservatorionazionalescreening.it/
10	Gezondheidsraad<br>(Health Council of Netherlands)	https://www.gezondheidsraad.nl/
11	Rijksinstituut voor Volksgezondheid en Milieu<br>(Dutch National Institute of Public Health and the Environment)	http://www.rivm.nl/

12	New Zealand National Screening Unit	https://www.nsu.govt.nz/
13	Ministerio de Sanidad, Servicios Sociales E Igualdad<br><br>(Spanish Ministry of Health, Social Services, and Equality)	http://www.msssi.gob.es/en/
14	Socialstyrelsen<br><br>(Swedish National Board of Health and Welfare)	http://www.socialstyrelsen.se/
15	UK National Screening Committee	https://legacyscreening.phe.org.uk/screening-recommendations.php /
16	U.S. Preventive Services Task Force	https://www.uspreventiveservicestaskforce.org/
**Referred to from initial organisations or experts**		
17	Australian Office of Population Health Genomics	http://www.genomics.health.wa.gov.au/
18	Canadian Organization for Rare Disorders	https://www.raredisorders.ca/
19	Canada: Canadian Agency for Drugs and Technologies in Health	https://www.cadth.ca/
20	Danish Statens Serum Institut	http://www.ssi.dk/
21	Finnish Office for Health Technology Assessment	http://www.inahta.org/
22	Società Italiana Studio Malattie Metaboliche Ereditarie<br><br>(Italian Society for the Study of Hereditary Metabolic Diseases and Neonatal Screening)	http://www.sismme.it/
23	Xunta de Galicia<br><br>(Department of Health of Galicia)	http://avalia-t.sergas.es/
24	Karolinska Universitetssjukhuset<br><br>(Karolinska University Hospital)	http://www.karolinska.se/
25	Advisory Committee on Heritable Disorders in Newborns and Children	https://www.hrsa.gov/advisorycommittees/mchbadvisory/heritabledisorders/
26	Human Genetics Society of Australasia	https://www.hgsa.org.au/

Appendix 2. Search terms for newborn blood spot screening

1	Newborn bloodspot, newborn blood spot	31	Hb S/ß-thalassemia (Hb S/ßTh)
2	Neonatal bloodspot, neonatal blood spot	32	Hb S/C disease (Hb S/C)
3	Guthrie test	33	Biotinidase deficiency (BIOT)
4	Bloodspot, blood spot	34	Cystic fibrosis (CF)
5	Heel prick	35	Classic galactosemia (GALT)
6	Newborn screening, new born screening	36	Methylmalonic acidemia with homocystinuria
7	Neonatal screening	37	Malonic academia
8	Propionic acidemia (PROP)	38	Isobutyrylglycinuria
9	Methylmalonic acidemia (mutase deficiency)(MUT)	39	2-Methylbutyrylglycinuria
10	Methylmalonic acidemia (mutase deficiency)(MUT)	40	3-Methylglutaconic aciduria
11	Isovaleric acidaemia (IVA)	41	2-Methyl-3-hydroxybutyric aciduria
12	3-Methylcrotonyl-CoA carboxylase deficiency (3MCC)	42	Short-chain acyl-CoA dehydrogenase deficiency
13	3-hydroxy 3-methyl glutaric aciduria (HMG)	43	Medium/short-chain L-3-hydroxyacl-CoA dehydrogenase deficiency
14	Multiple carboxylase deficiency (MCD)	44	Glutaric aciduria type II
15	ß-Ketothiolase deficiency (ßKT)	45	Medium-chain ketoacyl-CoA thiolase deficiency
16	Glutaric aciduria type I (GA1)	46	2,4 Dienoyl-CoA reductase deficiency
17	Carnitine uptake defect (CUD)	47	Carnitine palmitoyltransferase type I deficiency
18	Medium-chain acyl-CoA dehydrogenase deficiency (MCAD)	48	Carnitine palmitoyltransferase type II deficiency
19	Very long-chain acyl-CoA dehydrogenase deficiency (VLCAD)	49	Carnitine acylcarnitine translocasedeficiency
20	Long-chain L-3 hydroxyacyl-CoA dehydrogenase deficiency (LCHAD)	50	Argininemia
21	Trifunctional protein deficiency (TFP)	51	Citrullinemia, type II
22	Argininosuccinic acidemia (ASA)	52	Hypermethioninemia
23	Citrullinemia, type I (CIT)	53	Benign hyperphenylalaninemia
24	Maple syrup urine disease (MSUD)	54	Biopterin defect in cofactor biosynthesis
25	Homocystinuria (HCY)	55	Biopterin defect in cofactor regeneration
26	Classic phenylketonuria (PKU)	56	Tyrosinemia, type II
27	Tyrosinemia, type I (TYR1)	57	Tyrosinemia, type III
28	Primary congenital hypothyroidism (CH)	58	Hemoglobinopathies
29	Congenital adrenal hyperplasia (CAH)	59	Galactoepimerase deficiency
30	Sickle cell anemia (Hb SS)	60	Galactokinase deficiency

Appendix 3. Data Extraction Form: Blood spot review.

Reviewer:	
Country:	
Papers included:	
Were the review(s) independent?	

**Condition Specific**	Considered in a review document of any kind<br>Yes/No	Systematic review<br>Yes/No	Test accuracy score<br>1 to 5	Benefits and harms of early detection score<br>1 to 5	Overdiagnosis score<br>1 to 5	Recommended in review<br>Yes/No	Year	References
α1-Antitrypsin deficiency A1AT								
β-Ketothiolase deficiency BKT								
2-Methyl-3-hydroxybutyric aciduria 2M3HBA								
2-Methylbutyryl-CoA dehydrogenase deficiency 2MBG								
3-Hydroxy-3-methylglutaric aciduria HMG								
3-Methylcrotonyl-CoA carboxylase deficiency 3MCC								
3-Methylglutaconic aciduria 3MGA								
Adenosine deaminase deficiency ADA								
Arginine:glycine amidinotransferase deficiency AGAT								
Argininemia ARG								
Argininosuccinic acidemia ASA								
Benign hyperphenylalaninemia H-PHE								
Biliary atresia BIL								
Biotinidase deficiency BIOT								
Carbamoylphosphate synthetase deficiency CPS								
Carnitine palmitoyltransferase I deficiency (liver) [a]								
Carnitine palmitoyltransferase I deficiency (muscle) CPT IB [a]								
Carnitine palmitoyltransferase II deficiency CPT II								
Carnitine uptake defect CUD								
Carnitine/acylcarnitine translocase deficiency CACT								
Citrullinemia CIT								
Citrullinemia type II CIT II								
Classic galactosemia GALT								
Congenital adrenal hyperplasia (21-hydroxylase deficiency)								
Congenital cytomegalovirus infection CMV								
Congenital disorder of glycosylation type Ib CDG Ib								
Congenital hypothyroidism CH								
Congenital toxoplasmosis TOXO								
Creatine transport defect CR TRANS								
Cystic fibrosis CF								
Defects of biopterin cofactor biosynthesis BIOPT(BS)								
Defects of biopterin cofactor regeneration BIOPT(REG)								
Diabetes mellitus, insulin dependent IDDM								
Dienoyl-CoA reductase deficiency DE RED								
Duchenne and Becker muscular dystrophy DMD								
Fabry disease FABRY								
Familial hypercholesterolemia (heterozygote) FHC								
Fragile X syndrome FX								

**Condition Specific**	Considered in a review document of any kind Yes/No	Systematic review Yes/No	Test accuracy score 1 to 5	Benefits and harms of early detection score 1 to 5	Overdiagnosis score 1 to 5	Recommended in review Yes/No	Year	References
Galactokinase deficiency GALK								
Galactose epimerase deficiency GALE								
Glucose-6-phosphate dehydrogenase deficiency G6PD								
Glutaric acidemia type I GA I								
Glutaric acidemia type II GA2								
Guanidinoacetate methyltransferase deficiency GAMT								
Hearing loss HEAR								
Hemoglobin S/β-thalassemia Hb S/βTh								
Hemoglobin S/C disease Hb S/C								
Homocystinuria HCY								
Human HIV infection HIV								
Hurler-Scheie syndrome MPS-1H								
Hypermethioninemia MET								
Isobutyryl-CoA dehydrogenase deficiency IBG								
Isovaleric acidemia IVA								
Krabbe disease KRABBE								
Long-chainL-3-hydroxyacyl-CoA dehydrogenase deficiency								
Lysosomal storage diseases LSD								
Malonic acidemia MAL								
Maple syrup disease MSUD								
Medium/short-chainL-3-hydroxyacyl-CoA dehydrogenase								
Medium-chain acyl-CoA dehydrogenase deficiency MCAD								
Medium-chain ketoacyl-CoA thiolase deficiency MCKAT								
Methylmalonic acidemia (Cbl A,B) Cbl A,B								
Methylmalonic acidemia (Cbl C,D) Cbl C,D								
Methylmalonic acidemia (mutase deficiency) MUT								
Multiple carboxylase deficiency MCD								
Neonatal hyperbilirubinemia (kernicterus) HPRBIL								
Neuroblastoma NB								
Ornithine transcarbamylase deficiency OTC								
Phenylketonuria PKU								
Pompe disease POMPE								
Propionic acidemia PROP – NB in MMA section of report								
Severe combined immunodeficiency SCID								
Short-chain acyl-CoA dehydrogenase deficiency SCAD								
Sickle cell anemia (hemoglobin SS disease) Hb SS								
Smith-Lemli-Opitz syndrome SLO								
Trifunctional protein deficiency TFP								
Turner syndrome TURNER								
Tyrosinemia type I TYR I								
Tyrosinemia type II TYR II								
Tyrosinemia type III TYR III								
Variant hemoglobinopathies (including hemoglobin E) Var								
Very long-chain acyl-CoA dehydrogenase deficiency VLCAD								
Wilson disease WD								
X-linked adrenoleukodystrophy ALD								
Add unlisted conditions here…								

Reviewer Conclusion:

Appendix 4. Documentation excluded

No	Reference	Reason
1	The neonatal screening of PKU test [Available from: http://www.karolinska.se/for-vardgivare/kliniker-och-enheter-a-o/kliniker-och-enheter-a-o/karolinska-universitetslaboratoriet/cmms---centrum-for-medfodda-metabola-sjukdomar/information-om-nyfoddhetsscreeningen/ accessed 28 September 2015.	List of conditions currently included/considered for inclusion in programme
2	Newborn screening policy and guidelines. In: Prevention and Population Health, ed. Melbourne: Victorian Government Department of Health, 2011.	Description of current screening practice, policy or laws without reference to decision-making process
3	Australian Government Department of Health. Newborn bloodspot screening, Developing a policy framework for newborn bloodspot screening Australia: Australian Government Department of Health Undated [Available from: http://www.cancerscreening.gov.au/internet/screening/publishing.nsf/Content/newborn-bloodspot-screening accessed 18 September 2015.	Description of current screening practice, policy or laws without reference to decision-making process
4	Autti-Rämö I. HTA on neonatal screening for rare metabolic disorders faced misconceptions and blurred objectivity. *Orphanet Journal of Rare Diseases* 2012;7(Suppl 2):A17. doi: doi:10.1186/1750-1172-7-S2-A17	Duplication of included information
5	Autti-Rämö I, Laajalahti L, Koskinen H, et al. Vastasyntyneiden harvinaisten aineenvaihduntatautien seulonta. Helsinki: FinOHTAn, 2004.	Description of current screening practice, policy or laws without reference to decision-making process
6	Autti-Ramo I, Makela M. Screening for fetal abnormalities: from a health technology assessment report to a national statute. *International journal of technology assessment in health care* 2007;23(4):436-42. doi: 10.1017/S0266462307070663	No investigation of an included condition
7	Belgian Health Care Knowledge Centre. About the KCE Brussels: Belgian Health Care Knowledge Centre; Undated [Available from: http://kce.fgov.be/about-kce accessed 18 September 2015.	Description of organisation or study
8	Belgian Health Care Knowledge Centre. Study 2014-03 (GCP) Newborn blood spot screening for congenital diseases: scientific background Brussels: Belgian Health Care Knowledge Centre; Undated [Available from: http://kce.fgov.be/study-program/study-2014-03-gcp-newborn-blood-spot-screening-for-congenital-diseases-scientific-back accessed 18 September 2015.	Description of organisation or study

9	Bundesausschusses der Arzte. der Richtlinien des Bundesausschusses der Arzte und Krankenkassen uber die Fruherkennung von Krankheiten bei Kindern bis zur Vollendung der 4. Lebensjahres (Kinder-Richtlinien). Germany: Bundesausschusses der Arzte 1979.	Description of current screening practice, policy or laws without reference to decision-making process
10	Burgard P, Cornel M, Di Filippo F, et al. Report on the practices of newborn screening for rare disorders implemented in Member States of the European Union, Candidate, Potential Candidate and EFTA Countries. Europe: European Network of Experts on Newborn Screening, 2012.	Document not from national organisation
11	Cabinet Social Policy Committee. Newborn Metabolic Screening Programme Blood Spot Cards: New Policy and Governance Arrangements. New Zealand: Cabinet Social Policy Committee, 2011.	Document stating decision to change programme
12	Canadian Agency for Drugs and Technologies in Health. Newborn Screening for Disorders and Abnormalities in Canada. Canada: Canadian Agency for Drugs and Technologies in Health 2011.	Description of current screening practice, policy or laws without reference to decision-making process
13	Consejo Interterritorial. Criterios, acordados por el Consejo Interterritorial, que deben cumplir los CSUR para ser designados como de referencia del Sistema Nacional de Salud. España: Consejo Interterritorial, 2015.	Document not from national organisation
14	Côté B, Gosselin C. Pertinence d'élargir le programme de dépistage néonatal sanguin au Québec. Quebec: National Institute of Excellence in Health and Social Services, 2013.	Document not from national organisation
15	De Laet C. Number of conditions screened in Belgium: Personal communication, 2015.	List of conditions currently included/considered for inclusion in programme
16	des Bundesausschusses der Ärzte und Krankenkassen. Richtlinien des Bundesausschusses der Ärzte und Krankenkassen über die Früherkennung von Krankheiten bei Kindern bis zur Vollendung des 6. Lebensjahres („Kinder-Richtlinien"). Germany: des Bundesausschusses der Ärzte und Krankenkassen, 2011.	Description of current screening practice, policy or laws without reference to decision-making process
17	Gazzette Aufficiale. Disposizioni per la formazione del bilancio annuale e pluriennale dello Stato (Legge di stabilita' 2014). (13G00191) (GU Serie Generale n.302 del 27-12-2013 - Suppl. Ordinario n. 87). Italy: Gazzette Aufficiale, 2013.	Description of current screening practice, policy or laws without reference to decision-making process
18	Gemeinsamer Bundesausschuss. Bundesministerium für Gesundheit und Soziale Sicherung. Bekanntmachung eines Beschlusses des Gemeinsamen Bundesausschusses über eine Änderung der Richtlinien des Bundesausschusses der Ärzte und Krankenkassen über die Früherkennung von Krankheiten bei Kindern bis zur Vollendung des 6. Lebensjahres (Kinder-Richtlinien) zur Einführung des erweiterten Neugeborenen-Screenings. Germany: Gemeinsamer Bundesausschuss, 2004.	Document stating decision to change programme

19	Gemeinsamer Bundesausschuss. Beschlussbegründung über eine Änderung der Kinder-Richtlinien zur Einführung des erweiterten Neugeborenen-Screenings vom 21. Dezember 2004. Germany: Gemeinsamer Bundesausschuss, 2004.	Document stating decision to change programme
20	Gemeinsamer Bundesausschuss. Bekanntmachung eines Beschlusses des Gemeinsamen Bundesausschusses über eine Änderung der Kinder-Richtlinien: Anpassung des erweiterten Neugeborenen-Screenings an das Gendiagnostikgesetz (GenDG). Germany: Gemeinsamer Bundesausschuss, 2011.	Document stating decision to change programme
21	Gemeinsamer Bundesausschuss. Beschluss. des Gemeinsamen Bundesausschusses über die Einleitung des Beratungsverfahrens: Bewertung des Neugeborenen-Screenings zur Früherkennung der Tyrosinose Typ I mittels Tandem-Massenspektrometrie (TMS) – gemäß § 26 des Fünften Buches Sozialgesetzbuch (SGB V). Germany: Gemeinsamer Bundesausschuss, 2014.	Document stating decision to change programme
22	Gemeinsamer Bundesausschuss. Beschluss des Gemeinsamen Bundesausschusses über eine Beauftragung des Instituts für Qualität und Wirtschaftlichkeit im Gesundheitswesen: Bewertung des Neugeborenen-Screenings zur Früherkennung der Tyrosinämie Typ I mittels Tandem-Massenspektrometrie (TMS). Germany: Gemeinsamer Bundesausschuss; 2015.	Document stating decision to change programme
23	Gemeinsamer Bundesausschuss. Konkretisierung des Auftrags des Gemeinsamen Bundesausschusses an das Institut für Qualität und Wirtschaftlichkeit im Gesundheitswesen: Bewertung des Neugeborenen-Screenings zur Früherfassung der Tyrosinämie Typ I mittels Tandem-Massenspektrometrie (TMS). Germany: Gemeinsamer Bundesausschuss; 2015.	Contract
24	Knapp A, Metterville D, Kemper A, et al. Evidence Review: Critical Congenital Cyanotic Heart Disease. United States of America: MGH Center for Child and Adolescent Health Policy, 2010.	Not newborn bloodspot
25	Ministry of Health. Newborn Metabolic Screening: Policy framework. Wellington: Ministry of Health, 2011.	Description of current screening practice, policy or laws without reference to decision-making process
26	National Newborn Screening and Genetics Resource Center. Canada Status Report. Canada: Canadian Organization for Rare Disorders, 2008.	List of conditions currently included/considered for inclusion in programme
27	National Screening Unit. About the programme New Zealand: National Screening Unit; Undated [Available from: https://www.nsu.govt.nz/pregnancy-newborn-screening/newborn-metabolic-screening-programme-heel-prick-test/about-newborn accessed 28 September 2015.	Description of current screening practice, policy or laws without reference to decision-making process

28	National Screening Unit. Guidelines for practitioners providing services within the Newborn Metabolic Screening Programme in New Zealand. Wellington: National Screening Unit, Undated.	Description of current screening practice, policy or laws without reference to decision-making process
29	Nennstiel-Ratzel U, Genzel-Boroviczény O, Böhles H, et al. Newborn screening for congenital endocrinopathies metabolic disorders and endocrinopathies. Germany: AWMF online, 2011.	Document not from national organisation
30	Newborn metabolic screening programme National Metabolic Service. Appendix A. Summary assessment of 3MCCC and related disorders against the NHC Screening Criteria. New Zealand: National Screening Unit, 2014.	Duplication of included information
31	Newborn Screening Team. Newborn Screening. Information for parents concerning the early detection of congenital disorders in newborn babies program. Germany: Newborn Screening Team, Undated.	Patient information document
32	Office of Population Health Genomics. Newborn bloodspot screening policy framework Sydney: Government of Western Australia Department of Health Undated [Available from: http://www.genomics.health.wa.gov.au/nbspf/index.cfm#About accessed 18 September 2015.	Description of current screening practice, policy or laws without reference to decision-making process
33	Rijksinstituut voor Volksgezondheid en Milieu. De ziektes die de hielprik opspoort Bilthoven: Rijksinstituut voor Volksgezondheid en Milieu; Undated [Available from: http://www.rivm.nl/Onderwerpen/H/Hielprik/De_ziektes_die_de_hielprik_opspoort#alfa accessed 28 September 2015.	Patient information document
34	Ryall T. The New Policy and Governance Arrangements for Newborn Metabolic Screening Programme blood spot cards. New Zealand: The Ministry of Health, 2011.	Description of current screening practice, policy or laws without reference to decision-making process
35	Secretary's Advisory Committee on Heritable Disorders in Newborns and Children. Summary of Nominated Conditions to the Recommended Uniform Screening Panel (RUSP). United States of America: SACHDNC, 2014.	List of conditions currently included/considered for inclusion in programme
36	Secretary's Advisory Committee on Heritable Disorders in Newborns and Children. Recommended Uniform Screening Panel Core Conditions. United Stated of America: SACHDNC, 2016.	List of conditions currently included/considered for inclusion in programme
37	Società Italiana Studio Malattie Metaboliche Ereditarie. 23a Conferenza Nazionale sui Programmi di Screening Neonatale in Italia. Italy: SISSMME, 2014.	Description of current screening practice, policy or laws without reference to decision-making process
38	Società Italiana Studio Malattie Metaboliche Ereditarie. 24a Conferenza Nazionale sui Programmi di Screening Neonatale in Italia. Italy: SISSMME, 2015.	Description of current screening practice, policy or laws without reference to decision-making process

39	Statens Serum Institut. Sygdomme som indgår i screeningen Copenhagen: Statens Serum Institut; Undated [Available from: http://www.ssi.dk/Diagnostik/Center%20for%20Neonatal%20Screening/Sygdomme%20som%20indgaer%20i%20screeningen.aspx accessed 18 September 2015.	List of conditions currently included/considered for inclusion in programme
40	The Canadian Task Force on Preventive Health Care. Early Detection of Hyperthyroidism and Hypothyroidism in Adults and Screening of Newborns for Congenital Hypothyroidism. Canada: The Canadian Task Force on Preventive Health Care; 1990 [Available from: http://canadiantaskforce.ca/ctfphc-guidelines/1990-hyperthyroidism-and-hypothyroidism// accessed 18 September 2015.	Duplication of included information
41	The Canadian Task Force on Preventive Health Care. Screening for Cystic Fibrosis. Canada: The Canadian Task Force on Preventive Health Care; 1991 [Available from: http://canadiantaskforce.ca/ctfphc-guidelines/1991-cystic-fibrosis// accessed 18 September 2015.	Duplication of included information
